# Draft genome sequence of MBMR7, a *Bacillus altitudinis* phage recovered from the maize rhizosphere

**DOI:** 10.1128/mra.01461-25

**Published:** 2026-03-06

**Authors:** Mateus Belarmino, Armando Cavalcante Franco Dias, Thierry Alexandre Pellegrinetti, Elliot Watanabe Kitajima, Simone Raposo Cotta

**Affiliations:** 1 University of São Paulo, Luiz de Queiroz College of Agriculture (ESALQ), Piracicaba, São Paulo, Brazil; 2 University of São Paulo, Center for Nuclear Energy in Agriculture (CENA), Piracicaba, São Paulo, Brazil; 3 University of São Paulo, Center for Carbon Research in Tropical Agriculture (CCARBON), Piracicaba, São Paulo, Brazil; Queens College Department of Biology, Queens, New York, USA

**Keywords:** *Bacillus altitudinis *bacteriophage, rhizosphere soil, hellerevidae (Agatevirus)

## Abstract

In this report, we described the isolation and genomic characterization of *Bacillus altitudinis* phage MBMR7, isolated from a maize rhizosphere soil sample. The genome has a total length of 152,946 bp. By nucleotide similarity, MBMR7 is most closely related to *Bacillus* phages Bp8p-C and Bp8p-T.

## ANNOUNCEMENT

The activity of rhizosphere microorganisms directly contributes to plant and soil health, promoting nutrient cycling (1), suppressing pathogens (2), and degrading pollutants (3). *B. altitudinis* has shown potential to promote bioremediation (4) and plant growth (5). Phages can modulate the soil microbial community through their lytic and lysogenic infection cycles (6). Characterization of *B. altitudinis* phages can provide insights into the population dynamics of these bacteria in soil.

**Fig 1:**
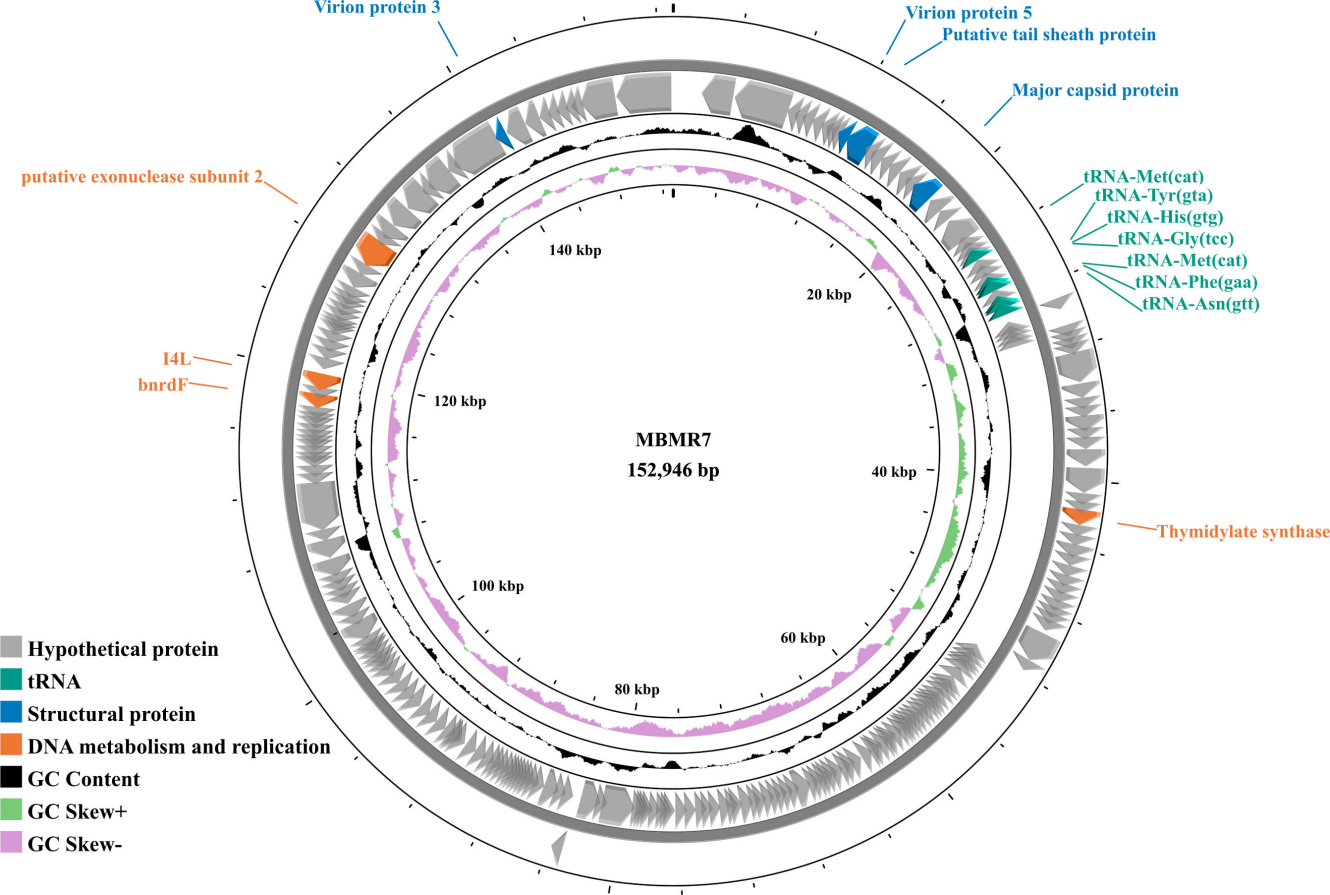
Phage MBMR7 genome map. Colored arrows indicate coding sequences potential function and transcriptional direction. Genes encoding hypothetical proteins are shown in gray. The inner rings represent the GC content and GC skew.

Phage MBMR7 was isolated from a maize rhizosphere soil sample collected in October 2025 in Piracicaba, São Paulo, Brazil (−22.703472, −47.634111) using a *B. altitudinis* strain isolated from the same area as a host. 0.5 g of soil was suspended in 5 mL of distilled water and vortexed for 10 min. The supernatant was collected and centrifuged (4°C) at 106 × *g* for 30 s and 6,500 × *g* for 15 min to remove soil debris and (7). The soil phage extract was filtered through a 0.22 µm filter and incubated in an amended Luria-Bertani (LB) broth (0.01% of CaCl_2_ and MgSO_4_ 1 M) containing the previously grown *B. altitudinis* culture at 25°C for 24 h. A 10^−2^ diluted aliquot of the phage-host suspension was then mixed with soft LB agar (0.6%) and plated using the agar double-layer method (8). The plates were incubated at 25°C for 24 h to allow plaque-forming units to develop. Three rounds of plaque purification were performed. DNA was extracted from phage lysate using the Wizard DNA clean-up kit (Promega, Madison, WI, USA) following the manufacturer’s instructions. DNA integrity was assessed by agarose gel (1%) electrophoresis and sequenced on an Illumina NextSeq 2000 platform. The library was constructed using the Illumina DNA Prep Kit (San Diego, CA, USA) according to the manufacturer’s instructions. A total of 39,923,584 paired-end reads (2 × 50 bp) were generated. The raw reads were quality-checked using FastQC v0.12.1 (9). Trimming was not performed because the read quality was high, and no adapters were present. The reads were assembled using Unicycler v0.4.8 (10). Sequence depth was determined using Bowtie2 v2.3.2 (11). 1,142 contigs were generated with an N50 of 116,066 bp and a coverage of 3,639×. Phage sequence identification was carried out with virSorter2 v1.0.1 (12), and fragment quality was evaluated using checkV v1.0.3 (13). Only one fragment classified as high-quality and complete was retained and considered full genome. Functional annotation was performed using Prokka v1.14.6 (14). A genome map was constructed with Proksee (15) (Fig. 1). Taxonomic assignment identified the phage as belonging to the *Herelleviridae* family using geNomad v1.9.0 (16). Phage lifestyle was predicted by PhageIA v1.0.2 (17). Default parameters were used for all software unless otherwise specified.

The phage MBMR7 genome has a total length of 152,946 bp, a GC content of 41,38%, a total of 223 genes, with an estimated completeness of 99.14% with no detectable contamination. No lysogeny-associated genes were detected, and a virulent lifestyle was predicted (99.93% confidence). Sequence similarity searches were performed using the BLASTn v.2.12.0 (18) against the ICTV (International Committee on Taxonomy of Viruses) database. MBMR7 shares a 91.20% nucleotide identity with phages Bp8p-C (NC_029121.1) and Bp8p-T (NC_047744.1) (query cover 87%), both placed in the Family *Herelleviridae* and the Genus *Agatevirus*.

## Data Availability

The draft genome of phage MBMR7 is available on GenBank under accession number PX674298. The raw sequencing data were deposited in the Sequence Read Archive (SRA) database under accession number SRX31257243.
